# The complete chloroplast genome of *Rungia pectinata* (Acanthaceae)

**DOI:** 10.1080/23802359.2019.1644216

**Published:** 2019-07-22

**Authors:** Zheli Lin, Sunan Huang, Yunfei Deng

**Affiliations:** a Key Laboratory of Plant Resources Conservation and Sustainable Utilization, South China Botanical Garden, Chinese Academy of Sciences, Guangzhou, People’s Republic of China;; b University of Chinese Academy of Sciences, Beijing, People’s Republic of China

**Keywords:** *Rungia pectinata*, chloroplast genome, phylogenetic, herbal medicine

## Abstract

*Rungia pectinata* is an important traditional Chinese herbal medicine from the family Acanthaceae. The complete chloroplast genome (cp genome) of the genus *Rungia* was sequenced for the first time. The cp genome of *R. pectinata* was 149,627 bp in length. It was consisted of a large single copy (LSC) region (81,976 bp) and a small single copy (SSC) region (16,626 bp), which were separated by two inverted repeats (IRs, 25,511 bp). This plastome contained 114 unique genes, including 80 protein-coding genes, 30 tRNA genes, and four rRNA genes. The overall GC content was 38.0%. Phylogenetic analysis of nine species in Acanthaceae was also conducted. This newly sequenced cp genome will be useful to further evolutionary studies, phylogenetic studies, and pharmacognostic identification in the genus *Rungia*.

*Rungia* Nees (Acanthaceae) is a genus comprising about 50 species distributed in the tropical and subtropical regions of the Old World (Hu et al. 2011; Mabberley 2017). *Rungia pectinata* (L.) Nees is a widespread species in Asia, the Chinese name of *R. pectinata* is “infant’s herb” as it is effective in treatment of infantile indigestion (Lo 1978). In this study, the complete chloroplast genome of the genus *Rungia* was sequenced for the first time, which will be useful to pharmacognostic identification in medicinal species of *Rungia*.

The complete chloroplast genome of *R. pectinata* was successfully assembled and characterized based on the Illumina pair-end (PE) sequencing data. The silica-gel dried leaves of *R. pectinata* were collected from China, Hainan Province, Baisha Xian, Nankai Xiang, Daoyin Cun (18°59′0.93″N, 109°19′45.88″E, 400 m). The voucher specimen (Z. L. Lin & Q. L. Wang 14022302) was deposited in the South China Botanical Garden Herbarium, Guangzhou, China. Total genomic DNA was isolated with a modified CTAB (Cetyl Trimethyl Ammonium Bromide) method (Doyle and Doyle 1987). Illumina Pair-end (PE) sequencing was performed on the Illumina HiSeq 2500 instruments at BGI-Wuhan. The sequenced clean PE reads were filtered using GetOrganelle pipeline (Jin et al. 2018) to get plastid-like reads. The filtered reads were assembled using SPAdes version 3.11.1 (Bankevich et al. 2012). The genome was automatically annotated using Plastid Genome Annotator (PGA) (Qu et al. 2019) coupled with manual corrections. The final complete plastome was deposited into GenBank (accession number MK946456).

The complete cp genome of *R. pectinata* was 149627 bp in length and presented a typical quadripartite structure including a large single copy (LSC) region (81,979 bp), a small single copy (SSC) region (16,626 bp), and two inverted repeat regions (IRs, 25,511 bp). The overall GC content of *R. pectinata* plastome was 38.0%. The *R. pectinata* plastome contained 114 different genes, including 80 protein-coding genes, 30 tRNA genes, and four rRNA genes. We also detected owing to the presence of internal stop codons, the gene ycf15 was identified as pseudogene in the plastome of *R. pectinata*.

To reconstruct the phylogeny of Acanthaceae, nine Acanthaceae cp genomes were included in the phylogenetic analysis; plastomes of two relative families were used as outgroups (see Figure 1 for details). The plastomes (each excluding one IR) of all the species were aligned using MAFFT (version 1.3.7) (Katoh and Standley 2013) implemented in Geneious v. 11.0.4 and adjusted manually when necessary. The maximum-likelihood (ML) phylogeny was reconstructed using RAxML version 8.0.0 (Stamatakis 2014).

**Figure 1.**
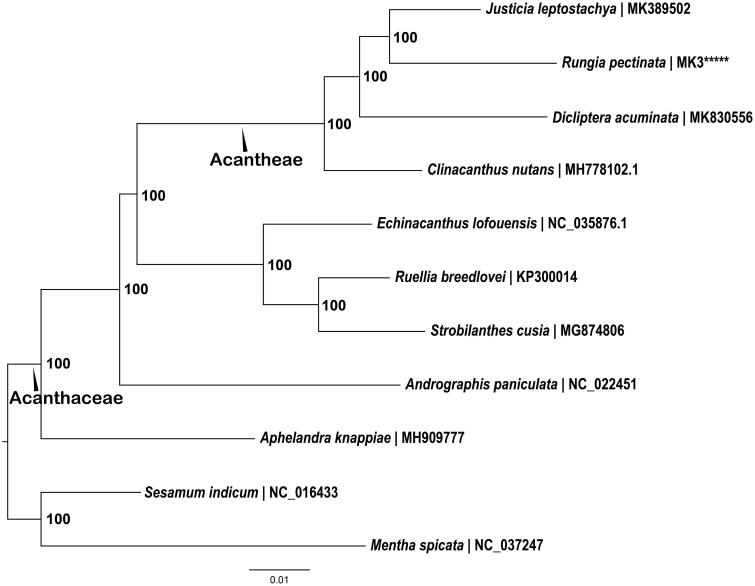
Maximum-likelihood (ML) phylogeny of Acanthaceae based on complete chloroplast genome sequences. Numbers at the right of nodes are bootstrap support values. GenBank accession numbers: *Andrographis paniculata* (NC_022451), *Ruellia breedlovei* (KP300014), *Strobilanthes cusia* (MG874806), *Echinacanthus lofouensis* (NC_035876.1), *Aphelandra knappiae* (MH909777), *Justicia leptostachya* (MK389502), *Clinacanthus nutans* (MH778102.1), *Dicliptera acuminata* (MK830556), *Sesamum indicum* (NC_016433), *Mentha spicata* (NC_037247), and *Rungia pectinata* (MK946456).

The present phylogenetic analyses strongly confirmed the monophyly of the family as previous reported (McDade et al. 2008) (Figure 1). Furthermore, all of the four Acantheae taxa formed a monophyletic group. Among the nine Acanthaceae species in this work, *R. pectinata* is most close to *Justicia leptostachya*, which all belong to Subtribe Justiciinae. All the nodes received 100% bootstrap support. The genome data in this paper can be subsequently used for phylogenetic studies in the genus *Rungia* and will contribute to further understanding of the phylogeny of Subtribe Justiciinae.
